# Lindsay & Blakiston’s Visiting List

**Published:** 1887-12

**Authors:** 


### Lindsay & Blakiston's Physician's Visiting List for 1888. Philadelphia: P. Blakiston, Son & Co.

The present issue is the thirty-seventh year of publication. It is divided in the usual way for the use of physicians of the regular school. Nevertheless, homœopathists can use it also.

It contains the usual valuable memoranda as heretofore.

